# Immune Thrombocytopenic Purpura and Ganglionic Tuberculosis: An Unlikely Link

**DOI:** 10.7759/cureus.62074

**Published:** 2024-06-10

**Authors:** Henrique Atalaia Barbacena, Miguel Esperança-Martins, Inês Matias Lopes, Leonor Branquinho, Patrícia Howell Monteiro

**Affiliations:** 1 Internal Medicine, Santa Maria’s Hospital, Local Health Unit of Santa Maria, Lisbon, PRT; 2 Pharmacology and Neurosciences, Faculty of Medicine, University of Lisbon, Lisbon, PRT; 3 Oncology, Santa Maria’s Hospital, Local Health Unit of Santa Maria, Lisbon, PRT; 4 Pathology, Santa Maria’s Hospital, Local Health Unit of Santa Maria, Lisbon, PRT

**Keywords:** immune mediated thrombocytopenic purpura (itp), extrapulmonary tuberculosis (eptb), ganglionic tuberculosis, lymphatic tuberculosis, thrombocytopenia

## Abstract

Tuberculosis (TB) is one of the leading infectious causes of morbidity and mortality worldwide. Either in its pulmonary (PTB) or extrapulmonary forms (EPTB), TB has a wide variety of manifestations, including hematological ones like thrombocytosis (especially in PTB) and thrombocytopenia (mainly with disseminated or miliary TB). Hematological manifestations are infrequently presenting features of TB, and within them, immune thrombocytopenic purpura (ITP)-associated TB is one of the rarest presenting features. We report a case of a 22-year-old woman with a diagnosis of ganglionic tuberculosis (GTB) presenting with ITP. The therapeutic approach was challenging and included the use, originally, of intravenous immunoglobulin 30 mg/day for five days and, posteriorly, of high-dose corticosteroids (dexamethasone 40 mg/day) and anti-tubercular therapy with satisfactory outcomes.

## Introduction

Tuberculosis (TB) is a multisystemic disease with myriad presentations and manifestations. It is the most common cause of infectious disease-related mortality worldwide [1]. Since 2020, TB incidence has been rising, with a total of 10.6 million people infected by the end of 2022 [2]. Individuals infected with Mycobacterium tuberculosis may develop signs and symptoms of the disease (active TB) or may have no clinical evidence of disease (latent TB infection (LTBI)). The diagnostic testing for LTBI is based on the interferon-γ release assay (IGRA) and tuberculin skin test (TST), while the diagnostic approach for active TB disease comprises, depending on whether it is pulmonary tuberculosis (PTB) or extrapulmonary tuberculosis (EPTB), acid-fast bacilli smear microscopy, nucleic acid amplification test, liquid and solid mycobacterial cultures, and histologic examination of specimens collected from suspected sites [3].

The relative frequency of EPTB is increasing [4,5]. It may be associated with a vast array of manifestations. The ganglionic or lymphatic (30-60% of the totality of EPTB cases) and pleural forms are the most frequent ones. When regarding ganglionic tuberculosis (GTB), the cervical ganglia are, by far, the most frequently affected, resulting in non-tender, elastic, and firm adenopathies. The identification of Koch’s bacillus (or acid-fast bacillus) on an aspiration product or a characteristic anatomical-pathological examination, such as epithelioid cell granulomas, caseous necrosis, and Langhans multinucleated giant cells, of a biopsied ganglion allows the formal diagnosis. The recommended therapeutic strategy is based on the classical six-month scheme with the association of isoniazid, rifampicin, ethambutol, and pyrazinamide [4].

Both EPTB and PTB are associated with a wide variety of hematological manifestations, such as anemia, leukocytosis, leukopenia, neutropenia, thrombocytopenia, pancytopenia, myelofibrosis, and hemophagocytic syndrome [4,5]. Normochromic normocytic anemia and a raised erythrocyte sedimentation rate are the most common hematological abnormalities in tuberculosis [4]. Thrombocytosis is seen mainly in PTB, and thrombocytopenia is commonly seen in disseminated or miliary TB. The pathophysiological mechanisms for thrombocytopenia in tuberculosis include a defect in platelet production, from bone marrow suppression associated with granulomatous infiltration; hemophagocytosis of all cell lineages in the bone marrow; side effects of antituberculous therapy, especially rifampicin and isoniazid; thrombotic thrombocytopenic purpura or disseminated intravascular coagulopathy; and immune-mediated platelet destruction [4]. When specifically considering TB-associated immune thrombocytopenic purpura (ITP), only 50 cases were reported in the literature between 1964 and 2016 [6-9], underlining the rarity of this event as a presenting feature of TB.

ITP is an auto-immune disorder characterized by a low platelet count and an increased risk of mucocutaneous bleeding. It is a heterogeneous syndrome with diverse pathological mechanisms, primarily mediated by autoantibodies. Platelets coated with immunoglobulin G (IgG) autoantibodies, normally directed against glycoprotein IIb/IIIa and/or glycoprotein Ib/IX and diverse other platelet determinants, undergo accelerated clearance through Fcγ receptors that are expressed by tissue macrophages in the spleen and liver. Alternatively, platelet production appears to be impaired subsequently due to intramedullary destruction of antibody-coated platelets by macrophages or inhibition of megakaryocytopoiesis [10]. 

ITP is classified as primary (defined by the International Working Group as a platelet count less than 100000/uL in the absence of other causes or underlying conditions [11]) or secondary. Secondary forms of the disease occur in association with several conditions, such as systemic lupus erythematosus (SLE), antiphospholipid syndrome (AFS), immunodeficiency states, lymphoproliferative disorders, infection with the human immunodeficiency virus and the hepatitis C virus, and therapy with drugs such as heparin and quinidine [10]. 

ITP remains a diagnosis of exclusion, and misdiagnosis is common, even by experienced hematologists [11]. It is important to emphasize that the direct assay for the measurement of platelet-bound antibodies has an estimated sensitivity of 49 to 66%, an estimated specificity of 78 to 92%, and an estimated positive predictive value of 80 to 83% and, therefore, cannot be used to confirm or rule out the diagnosis [11]. 

The therapeutic options serve the purposes of either rapidly, but transiently, raising the platelet count level to maintain a stable, hemostatic platelet count or, finally, achieving remission. First-line treatments include corticosteroids, intravenous immune globulin (IVIG), or Rh(D) immune globulin [12-13]. The urgency of a platelet count response and the patient's comorbidities and preferences should guide which treatment should be prescribed [12].

## Case presentation

A 22-year-old African-Portuguese woman was admitted to the emergency department with a six-day history of epistaxis and gingivorrhagia. Concomitantly, she noted two weeks earlier the enlargement of nodular tender swellings in the left cervical region, with progressive enlargement. She denied involuntary weight loss, fever, night sweats, previous episodes of epistaxis, hemoptysis, hematuria, hematemesis, melena, hematochezia, any skin changes compatible with purpura, features compatible with subconjunctival hemorrhage, any other organ-specific symptoms, recent drug exposure, and travel history. The patient had no significant past medical history. Family history was not relevant, and the patient's family was not sick. The patient was born and grew up in Guinea-Bissau, arriving in Portugal at the age of 17. 

At the first examination, the patient presented with normal vital signs, a normal body temperature, and an oxygen saturation of 99% in room air. Bilateral elastic cervical adenopathies were noted, slightly tender to palpation. Oral hematomas were also noted on the lips. The skin had no lesions compatible with purpura, and the eyes had no signs suggesting subconjunctival hemorrhage.

Initial assessment showed (Table 1) a complete blood count with severe thrombocytopenia (3x10^9/L), normocytic/normochromic anemia, a hemoglobin value of 11.4 g/dL, and no changes in white cell count; an erythrocyte sedimentation rate of 72 mm/h; elevated C-reactive protein (3.05 mg/dL); and lactate dehydrogenase (LDH) of 331 U/L. The remaining biochemistry profile and coagulation tests were normal. The chest radiograph showed no significant findings.

**Table 1:** Blood work evaluation during hospital stay During admission, we can observe the evolution of platelet counts. The patient received only a platelet transfusion (PT) as support therapy during the first and third days of admission. Throughout this period, platelet counts only slightly improved. On the third day, IVIg was started, and by the fifth day of therapy (D7 adm), the platelet count was normal, and the patient was discharged. One week after discharge, the patient was observed in an internal medicine appointment with a recurrence of severe thrombocytopenia (2x10^9/L) and was readmitted to the ward. Given the histological lymph node analysis and IGRA results at readmission, the patient was initiated on anti-tuberculosis and high-dose IV corticosteroids therapy. From D2 readm to D10 readm, platelet count swiftly and steadily normalized. On a second observation in the ambulatory clinic, the patient had no symptoms, and the platelets remained normal. Adm: admission; aTB: antituberculosis therapy; HDCS: high dose IV corticosteroids; IM: internal medicine; IVIg: IV immunoglobulin; OCS: oral corticosteroids; PT: platelet transfusion; Readm: readmission

Laboratory parameter	Reference value and units	D1 adm(PT)	D2 adm(PT)	D3 adm(IVIg)	D7 adm (IVIg; 1st discharge)	IM appointment	D2 readm(HDCS + aTB)	D5 readm(HDCS + aTB)	D10 readm (OCS + aTB; 2nd discharge)	IM appointment(OCS + aTB)
Hemoglobin	13-16g/dL	11.4	10.8	11.1	10.2	9.6	9.6	11.0	9.6	9.9
Leukocyte count	4.0 - 11.0x10^9^/L	7.81	7.36	5.85	5.82	8.60	16.47	8.10	6.83	8.47
Platelet count	150 - 450 x10^9^/L	3	6	13	161	2	21	291	164	210
International normalized ratio	1.0	1.17	1.22	-	-	1.18	-	-	-	-
Activated partial thromboplastin time (aPTT)	29.0 seg	32.0	27.7	-	-	28.4	-	-	-	-
Fibrinogen	200-400 mg/dL	474	433	-	-	-	-	-	-	-
D-dimers	<0.5 ug/mL	0.42	-	-	-	-	-	-	-	-
C-reactive protein	<0.5 mg/dL	3.05	2.78	-	1.41	3.78	4.30	0.25	0.28	0.3
Lactate dehydrogenase	100-250 U/L	331	326	-	243	243	322	232	-	236
Haptoglobin	30-200 mg/dL	332	-	-	-	-	-	-		-

The patient was admitted to an internal medicine ward, and initial support therapy was pursued with the transfusion of two platelet concentrates, with only a marginal elevation of the platelet count to 13x10^9/L. An extensive immune-serological study was performed, showing: positive anti-platelet antibodies (GPIb-IX); positive antinuclear antibodies (ANA) with a titre of 1:160; positive anti-Sjögren-syndrome-related antigen A/Ro (anti-SSA/Ro) antibodies with a titre of 265.8 UI/mL; no complement consumption, with normal values of CH50, C3, and C4; negative anti-double-stranded-DNA (anti-dsDNA) antibodies; anti-Sjögren-syndrome-related antigen B/La; anti-Smith (anti-Sm); anti-ribonucleoprotein (anti-RNP); anti-Jo1; anti-neutrophil cytoplasmic antibodies (ANCA); anti‐topoisomerase I (anti-Scl-70); lupus anticoagulant; anti-cardiolipin; and anti-beta-2 glycoprotein 1 antibody. Serological testing for infectious causes showed: negative human immunodeficiency virus (HIV), Ebstein-Barr virus (EBV), cytomegalovirus (CMV), parvovirus B19, hepatitis B virus (HBV), hepatitis C virus (HCV), syphilis, Leishmania spp., Brucella spp., Coxiella burnetti, Borrelia burgdorferi, and Bartonella henselae.

Given the absence of an adequate response, the presence of hemorrhagic symptoms, and the positivity of antiplatelet antibodies, on the third day of admission, it was decided to start intravenous immunoglobulin (IVIg) at 30 mg/day for five days, hence the higher probability of corticosteroids distorting the histological architecture of adenopathies, compromising the final result. Before the beginning of this therapy with IVIg, a fine needle aspiration biopsy of anterior cervical adenopathy was performed, and the immunophenotyping studies of the ganglionic aspirate were normal, excluding a hematological primary cause for ITP.

The use of IVIg allowed for a significant increase in platelet count, making it possible for a bone marrow biopsy and a cervical lymphadenectomy on the fourth day of therapy. 

On the fifth day of therapy, with an increase in the platelet count to 161x10^9/L (Table 1), the patient was discharged with an internal medicine appointment, awaiting the results of the bone marrow biopsy and the lymph node histological analysis. 

One week after hospital discharge, the patient was evaluated in the ambulatory clinic, mentioning a recurrence of epistaxis and gingivorrhagia and new complaints of menorrhagia, although the patient was under progestative anti-contraceptive therapy, night sweats, and fever. Blood work revealed a recurrence of severe thrombocytopenia (2x10^9/L) with a still slightly elevated C-reactive protein (3.78 mg/dL), with no other findings worth mentioning (Table 1). 

**Figure 1:**
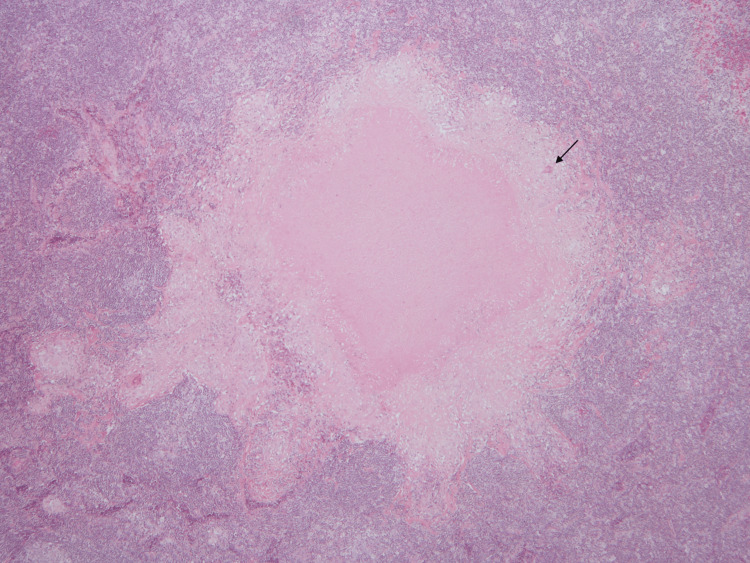
Lymph node histology (x10 ampliation) Lymph node with architectural distortion due to the presence of epithelioid granulomas with central caseous necrosis. The Langhans cells are visible (black arrow).

**Figure 2:**
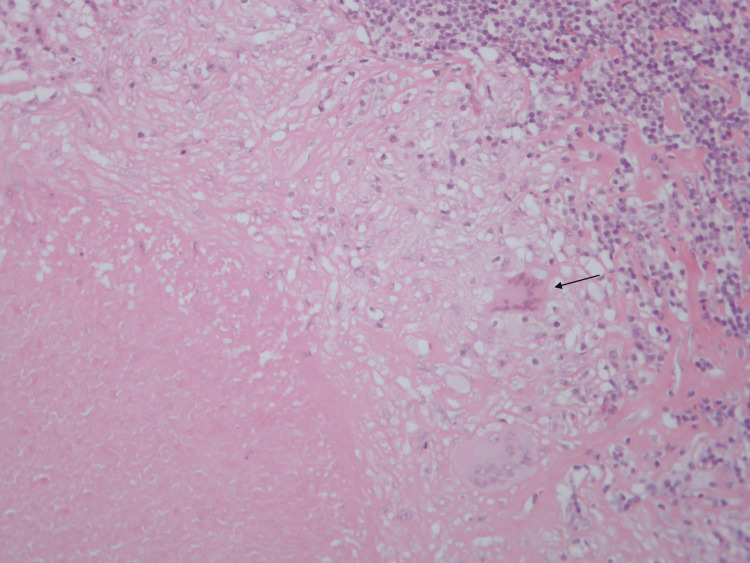
Lymph node histology (x20 ampliation) Multinucleated cells of Langhans are seen (black arrow). Although not pathognomonic, these cells are frequently encountered in tuberculosis infections.

The patient was readmitted to the ward. A bone marrow biopsy showed normal cellularity, a myeloid:erythroid ratio of 6:1, a diminished number of megakaryocytes, and normal remaining features. The anatomical-pathological examination of the excised lymph node revealed the presence of coalescent epithelioid cell granulomas with suppurative caseous necrosis and multinucleated foreign-body and Langhans giant cells (Figures 1, 2). Blood samples were collected for mycobacterial blood cultures, the IGRA test, and the determination of angiotensin-converting enzyme levels. The blood cultures for anaerobes and aerobes, as well as prolonged cultures for mycobacteria, were all negative. The IGRA test was positive, and the angiotensin-converting enzyme levels were normal. A diagnosis of an ITP secondary to GTB was considered very likely.

The case was discussed among the internal medicine, hematology, and infectious diseases teams. Regarding the diagnosis of an ITP secondary to GTB, and based on the protocol used in our center, it was decided to start corticosteroids, namely dexamethasone 40 mg/day for four days and posteriorly prednisolone 1 mg/kg/day with a progressive tapering schedule. Simultaneously, anti-tuberculous therapy with isoniazid 300 mg/day, pyrazinamide 1500 mg/day, ethambutol 1200 mg/day, pyridoxine 40 mg/day, and rifampicin 600 mg/day was started. 

Clinical improvement was noted, with resolution of hemorrhagic manifestations, followed by an increase in platelet count, with a value of 164x10^9/L at the time of discharge (Table 1). 

An internal medicine appointment was arranged after discharge, and the patient was also referred to the Reference Center for Tuberculosis Treatment, where patients with a suspected or confirmed diagnosis of tuberculosis are followed up and treated in Portugal. The patient finished her tapering schedule of corticosteroids and continued the anti-tuberculous drugs previously prescribed. Blood work showed a consistent and increasing value of platelet count (Table 1), remaining stable even after six weeks of corticosteroid tapering.

## Discussion

The conjugation of the patient's demographic data (an African-Portuguese young woman originally from Guinea-Bissau), its original clinical picture (epistaxis, gingivorrhagia, and the progressive enlargement of nodular tender unilateral cervical swellings), and the first blood test results (severe thrombocytopenia, discrete normocytic/normochromic anemia, and a slightly elevated LDH value) made the diagnosis of a secondary ITP, most likely a lymphoproliferative disease, even though infectious and auto-immune disorders couldn’t be ruled out by that time, relatively likely. Although the positivity of anti-platelet antibodies is not diagnostic and their absence cannot be used to rule out the diagnosis, they have a positive predictive value of 80-83% [11], and together with the increase in platelet count after immunosuppressive therapy with IVIg and its recurrence after the end of such therapy, strongly support the diagnosis of ITP. The immune-serological studies, bone marrow biopsy, and histological examination of the lymph node were crucial to confirm and rule out secondary causes of ITP since primary ITP remains a diagnosis of exclusion [4,6,8,11].

Albeit the initial diagnostic suspicion of ITP secondary to a lymphoproliferative disease, namely due to the age and race of the patient, together with the progressive enlargement of adenopathies, LDH elevation, and the absence of epidemiological or familial clues to infectious or autoimmune disorders, blood work showed consistently no changes in white cells, the immunophenotyping of the ganglionic aspirate was normal, and, particularly, the bone marrow biopsy had no relevant findings, making this hypothesis unlikely. The new clinical features, such as night sweats and fever without respiratory complaints, in combination with a normal chest radiograph and the anatomical-pathological characteristics of the excised lymph node, namely the coalescent epithelioid cell granulomas, suppurative caseous necrosis, and multinucleated foreign-body and Langhans giant cells, together with the positive IGRA test, allowed us to establish the diagnosis of ganglionic TB. The negative immune-serological studies discarded the possibility of other infectious or auto-immune disorders as the cause of the ITP. Therefore, a diagnosis of an ITP secondary to ganglionic TB was considered very likely.

A search of the literature available on TB-associated ITP identified circa 55 cases published between 1964 and 2020 [6-9]. Weber et al. [6] reviewed the 50 cases of TB-associated ITP that have been published until 2016 and established four case definitions of the likelihood of causality. Highly suggestive: recovery of platelet count not achieved by first-line ITP treatment alone but by anti-tuberculosis treatment either as a primary therapy without immune modulation or started after failure of the ITP regimen; suggestive: recovery of platelet count achieved by simultaneous administration of first-line ITP therapy and anti-tuberculosis treatment; possible: non-homogeneous group, temporal association is present, but causality is either unlikely or not very suggestive; unlikely: unlikely cause association. Only 34 of the original 50 cases were considered highly suggestive, and a specific analysis of those cases was performed: gender was evenly distributed, although a relative predominance of females was seen; age ranged from four to 74 years old; initial platelet count ranged between <1000/uL and 46000/uL; hemorrhagic symptoms were present in most cases (27/28 cases had bleeding symptoms, with skin manifestations being the most common; mucosal bleeding, hematuria, and epistaxis were also reported); 21 cases were microbiologically confirmed and six cases were histologically confirmed; 41% of patients had PTB, 21% had disseminated TB, and the remaining had EPTB at various sites, and no exclusive associations between TB site and ITP were noted [6].

The pathophysiology of secondary ITP associated with TB isn’t completely evident. Suggested mechanisms include the production of anti-platelet antibodies and molecular mimicry during a regular immune response to TB. However, the detection of antibodies against platelet antigens was not frequently reported in these cases [6-9].

In the majority of these, first-line therapy for ITP, such as steroids or other immune-modulating agents, was started prior to anti-tuberculosis treatment as the diagnosis of TB was only established later. A minor increase in platelet count was achieved under immune-modulation medication; therapy extension or switching to anti-tuberculosis treatment resulted in a significant increase in platelet count. Considering the therapeutic experience of TB-associated ITP, the beneficial effect of immune modulatory treatment remains unclear, as platelet count recovery is achievable by anti-tuberculosis treatment alone [6,12,13]. 

In our case, as previously mentioned, the suspicion of an ITP was strongly supported by the positivity of the antiplatelet antibodies. Although its presence is not diagnostic, nonetheless they have a significant positive predictive value, and the increase of the platelet count with the immunosuppressive therapy, together with its rebound after discontinuation of such therapy, strengthens the ITP hypothesis. All studies conducted allowed for the elimination of other etiologies, namely immune serological studies, infectious serologies, bone marrow analysis, and lymph node phenotyping. 

The clinical features of the latter are obvious, namely night sweats and fever, in combination with a normal chest radiograph and absence of respiratory symptoms (excluding PTB), the anatomical-pathological characteristics of the excised lymph node (namely the necrotic caseous granulomas with giant multinucleated Langhans cells; Figures 1, 2), and finally, the positive IGRA test, which allowed us to establish the diagnosis of GTB. 

Regarding the case definitions of the likelihood of causality listed by Weber et al. [6], the causality between ITP and GTB in our case can be defined as “highly suggestive,” since the recovery of the platelet count was not achieved by a first-line ITP treatment alone (IVIg), but by anti-tuberculosis treatment in combination with corticosteroids.

Although the IVIg effect will wane in about three to four weeks, we must note that the patient presented to the internal medicine appointment only seven days after discharge, with a platelet count that goes from 161x10^9/L at discharge to 2x10^9/L (Table 1). This means the IVIg effect waned off much before the expected time period, an event unlikely given the drug's half-life. 

Considering the characteristics of the 34 cases whose causality was considered highly suggestive and suggestive, that were also analyzed by Weber et al: our patient was female, 22 years old, with hemorrhagic symptoms (mucosal bleeding and epistaxis, like in the majority of those cases), with a histological confirmation of tuberculosis (only a minority of those cases had this type of confirmation), and with a highly suggestive causality between ITP and EPT (like in 38% of those cases), specifically GTB. 

Although the case classifies as a highly suggestive causality between ITP and GTB, we cannot ignore the positivity of ANA and anti-SSA/Ro antibodies. ITP is known to be associated with autoimmune diseases such as SLE and Sjögren syndrome (SS); however, the patient denied any rheumatic symptoms, either spontaneously or upon questioning. Clinical examination did not show any signs of articular inflammation or other suggestive features of an autoimmune background. Regarding ANA antibodies, they are somewhat unspecific and can be present in many other diseases and states other than autoimmune disorders. Hyoung Im et al. [14] searched in a retrospective fashion for the association between ANA positivity and infectious diseases, and by far the most prominent association was with Mycobacterium tuberculosis infections. The authors concluded that ANA positivity can be associated with infectious diseases, particularly intracellular infections, such as TB. Shen et al. [15] also analyzed in a retrospective fashion the prevalence of antibodies in TB and found that one-third of patients with active TB had elevated antibody levels. A significant relationship between anticardiolipin IgG and anti-Scl-70 was established. Additionally, anti-SSA/Ro was also found in TB patients, although not in a statistically significant fashion. The authors postulated that these antibodies do not have a pathognomonic role since their presence does not alter either the clinical or radiographic presentation of the disease and their presence could be related to a break in self-tolerance, where fragments of mixed self-antigens and pathogen-antigens induce immune responses, as in a mode of epitope spread in chronic inflammation states such as TB [15]. The authors also stated that in the majority of the cases, antibody positivity disappeared after TB treatment, hence a causal relationship between the two phenomena [15]. Thus, in our case, the antibody presence is more probably due to the chronic inflammation and infection caused by GTB than to a real autoimmune background, given the absence of clinical signs and symptoms.

## Conclusions

ITP is a rare condition, more frequently secondary to other diseases, and the differential diagnosis is extensive. TB, although one of the most frequent chronic infections in the world, has a multitude of manifestations. GTB is one of the rarer EPTB manifestations, affecting mainly children and women, with a predominance in Asian countries. Hematological manifestations associated with TB are common; however, the association of ITP with TB is a rare event. In our case, we found an association between GTB and ITP, making this case unique and underscoring the importance of considering TB as a possible cause of secondary ITP. The diagnosis of GTB is established given the positive IGRA, the anatomical-pathological aspects highly suggestive of TB (central caseous necrosis granulomas and multinucleated Langhans cells), together with the epidemiological data (Guinea-Bissau), and the exclusion of other causes for secondary ITP (namely other infectious stimuli and autoimmune diseases). Causality between GTB and ITP is reinforced by the poorer efficacy of immune-suppressive therapies, in our case IVIg, the recurrence of severe thrombocytopenia when immune therapy was suspended, and the swift and steady normalization of platelet count when anti-tuberculosis treatments were used, even after corticosteroids discontinuation, making them the mainstay treatment of ITP associated with TB.

## References

[1] Herchline T, Amorosa J (2024). Tuberculosis (TB). https://emedicine.medscape.com/article/230802-overview?form=fpf.

[2] (2023). Global Tuberculosis Report 2023. https://iris.who.int/bitstream/handle/10665/373828/9789240083851-eng.pdf?sequence=1.

[3] Lewinsohn DM, Leonard MK, LoBue PA (2017). Official American Thoracic Society/Infectious Diseases Society of America/Centers for Disease Control and Prevention clinical practice guidelines: diagnosis of tuberculosis in adults and children. Clin Infect Dis.

[4] Nasa P, Juneja D, Sehra S, Singh HK, Prasad DB (2019). Immune thrombocytopenic purpura in a patient with disseminated tuberculosis: an unusual presentation. Int J Mycobacteriol.

[5] Ketata W, Rekik WK, Ayadi H, Kammoun S (2015). Extrapulmonary tuberculosis (Article in French). Rev Pneumol Clin.

[6] Weber SF, Bélard S, Rai S, Reddy R, Belurkar S, Saravu K (2017). Immune thrombocytopenia secondary to tuberculosis: a case and review of literature. Int J Tuberc Lung Dis.

[7] Dagaonkar RS, Udwadia ZF (2012). Disseminated tuberculosis with immune thrombocytopenic purpura. Lung India.

[8] Srividya G, Nikhila GP, Kaushik AV, Jayachandran K (2014). Immune thrombocytopenia in tuberculosis: causal or coincidental?. J Glob Infect Dis.

[9] Rama Krishna M, Gottam US, Mahendra N (2019). Disseminated tuberculosis with severe immune thrombocytopenia. Respir Med Case Rep.

[10] Cines DB, Blanchette VS (2002). Immune thrombocytopenic purpura. N Engl J Med.

[11] Rodeghiero F, Stasi R, Gernsheimer T (2009). Standardization of terminology, definitions and outcome criteria in immune thrombocytopenic purpura of adults and children: report from an international working group. Blood.

[12] Neunert C, Terrell DR, Arnold DM (2019). American Society of Hematology 2019 guidelines for immune thrombocytopenia. Blood Adv.

[13] Mithoowani S, Arnold DM (2019). First-line therapy for immune thrombocytopenia. Hamostaseologie.

[14] Im JH, Chung MH, Park YK, Kwon HY, Baek JH, Lee SY, Lee JS (2020). Antinuclear antibodies in infectious diseases. Infect Dis (Lond).

[15] Shen CY, Hsieh SC, Yu CL, Wang JY, Lee LN, Yu CJ (2013). Autoantibody prevalence in active tuberculosis: reactive or pathognomonic?. BMJ Open.

